# Environmental footprint of peritoneal dialysis in Europe: a comparative life cycle assessment across four European centres

**DOI:** 10.1093/ckj/sfag156

**Published:** 2026-05-13

**Authors:** Abass Fehintola, Zuzanna Jakubowska, Malgorzata Kepska-Dzilinska, Jolanta Malyszko, Guus Crooijmans, Ootjers Thomas, James Larkin, Giulia Ligabue, Gaetano Alfano, Gabriele Donati, Rodrigo MartínezCadenas, Aycan Yasar, Belén Sanz, Gonzalez M de Rivera, Lucia Cordero, Julia Audije-Gil, María Dolores Arenas, Ingeborg Steinbach, Marta Arias, Alberto Ortiz, Karin Gerritsen, Brett Duane

**Affiliations:** School of Dental, Child and Public Health,Trinity College Dublin, Dublin, Ireland; Department of Nephrology, Dialysis and Internal Medicine, Medical University of Warsaw (WUM), Warsaw, Poland; Department of Nephrology, Dialysis and Internal Medicine, Medical University of Warsaw (WUM), Warsaw, Poland; Department of Nephrology, Dialysis and Internal Medicine, Medical University of Warsaw (WUM), Warsaw, Poland; Department of Nephrology and Hypertension, University Medical Centre Utrecht (UMCU), Utrecht, Netherlands; Department of Nephrology and Hypertension, University Medical Centre Utrecht (UMCU), Utrecht, Netherlands; School of Dental, Child and Public Health, Trinity College Dublin, Dublin, Ireland; Nephrology Dialysis and Kidney Transplant Unit, AOU Policlinico di Modena, Modena, Italy; Nephrology Dialysis and Kidney Transplant Unit, AOU Policlinico di Modena, Modena, Italy; Nephrology Dialysis and Kidney Transplant Unit, AOU Policlinico di Modena, Modena, Italy; Department of Nephrology and Hypertension, Health Research Institute-Fundación Jiménez Díaz University Hospital, Universidad Autónoma de Madrid (IIS-FJD, UAM), Madrid, Spain; Centre for Sustainable Healthcare, Oxford, UK; Unidad de investigación, Fundación Renal Española, Madrid, Spain; Department of Nephrology and Hypertension, Health Research Institute-Fundación Jiménez Díaz University Hospital, Universidad Autónoma de Madrid (IIS-FJD, UAM), Madrid, Spain; Unidad de investigación, Fundación Renal Española, Madrid, Spain; Unidad de investigación, Fundación Renal Española, Madrid, Spain; Unidad de investigación, Fundación Renal Española, Madrid, Spain; Centre for Sustainable Healthcare, Oxford, UK; Nephrology and Renal Transplantation Unit, Hospital Clinic Barcelona, Barcelona, Spain; Department of Nephrology and Hypertension, Health Research Institute-Fundación Jiménez Díaz University Hospital, Universidad Autónoma de Madrid (IIS-FJD, UAM), Madrid, Spain; RICORS2040-renal, Madrid, Spain; Departamento de Medicina, Facultad de Medicina, Universidad Autónoma de Madrid, Madrid, Spain; Department of Nephrology and Hypertension, University Medical Centre Utrecht (UMCU), Utrecht, Netherlands; School of Dental, Child and Public Health,Trinity College Dublin, Dublin, Ireland

**Keywords:** chronic kidney disease, environmental impact, life cycle assessment, peritoneal dialysis, sustainability

## Abstract

**Introduction:**

Peritoneal dialysis (PD) is a home-based renal replacement therapy offering clinical and quality-of-life advantages, but it also poses environmental challenges due to resource use and waste generation. This study compares the environmental impacts of PD across four European centres to identify regional sustainability differences and evaluate the footprint of continuous ambulatory (CAPD), automated (APD), and incremental (iPD) PD.

**Methods:**

A cradle-to-grave life cycle assessment (LCA) was conducted for one year of PD treatment per patient using OpenLCA software and the EcoInvent database across four clinical centres; Madrid, Warsaw, Utrecht, Modena. Data were collected on material use, energy and water consumption, transportation, and waste disposal. Key indicators included global warming potential (GWP), energy use, water footprint. The environmental performance of each centre and dialysis modality was assessed.

**Results:**

Environmental impacts varied significantly between centres ranging from GWP 3381 kg CO₂-eq to 1736 kg, reflecting differences in energy mix and efficiency. Energy consumption ranged from 54,710 MJ to 28,894 MJ and water footprint from 6631 m³ to 854 m³. Across all centres, CAPD and APD had higher environmental burdens than iPD. iPD reduced emissions by up to 50% and had a lower-impact profile.

**Conclusions:**

PD displays substantial environmental impact variability across centres, driven by differences in material consumption, waste management, energy sourcing, and clinical protocols. Cleaner energy profiles, effective waste management, and reduced reliance on single-use consumables may lower environmental burdens. These findings underscore the importance of integrating environmental performance into clinical decision-making and health system planning.

## Graphical Abstract

**Graphical Abstract:**
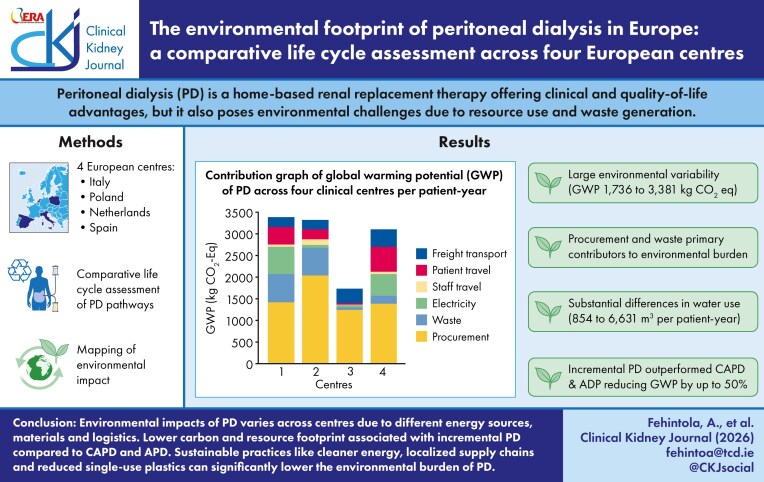

## INTRODUCTION

Kidney disease affects over 850 million individuals globally, presenting a significant burden on healthcare systems [1]. Kidney failure requires renal replacement therapies (RRTs) such as dialysis or kidney transplantation to sustain life. Peritoneal dialysis (PD) offers home-based treatment, enhanced patient autonomy, flexibility, and quality of life compared to in-centre haemodialysis (HD) [1]. The healthcare sector contributes significantly to global resource consumption and greenhouse gas emissions, and the European Renal Association (ERA) has consistently placed environmental sustainability on the nephrology agenda, calling for evidence-based assessments of the carbon and resource footprint of kidney care pathways [2, 3]. PD, like other medical interventions, involves resource-intensive processes such as the production and disposal of single-use plastics, energy consumption for automated dialysis machines, and patient travel for consultations [4], highlighting the need for systematic evaluation and intervention.

Within this context, life cycle assessment (LCA) provides a rigorous, standardized methodology to quantify environmental burdens across the full treatment pathway and to identify the highest-impact stages amenable to targeted intervention. LCA has been successfully applied to assess different HD practices [5–7].

By examining the impacts of PD from resource extraction to end-of-life disposal, LCA enables the identification of high-impact stages and facilitates the development of targeted strategies for sustainability. While existing studies have provided valuable insights into the environmental footprint of PD, they are often limited to single clinical centres, which may not account for regional variations in energy infrastructure, waste management practices, and clinical protocols [3, 8].

The two main modalities of PD are continuous ambulatory peritoneal dialysis (CAPD) and automated peritoneal dialysis (APD) [9–11]. They offer distinct clinical and logistical advantages but differ in their environmental impacts. CAPD relies on manual exchanges of dialysis fluid, while APD requires a machine for overnight exchanges, leading to increased energy consumption. Incremental PD (iPD), in which patients commence with a sub-full-dose prescription, represents a third modality of growing clinical and environmental interest [12].

This study conducted a comparative LCA of PD across four European clinical centres: Modena (Italy), Utrecht (Netherlands), Warsaw (Poland), and Madrid (Spain). These centres were chosen to represent diverse regional contexts, reflecting variations in energy sources, healthcare policies, and environmental practices. By analysing the environmental impacts of PD treatments in these locations, this study seeks to provide a comprehensive understanding of the regional factors influencing sustainability in kidney healthcare. The findings would support environmentally conscious clinical decision-making and contribute to the ERA’s broader sustainability initiative. Specific objectives were to identify key contributors to the environmental footprint of PD in each centre, examine regional variation in impacts based on energy, waste, and clinical practice and propose targeted strategies for improving the sustainability of PD pathways.

## METHODOLOGY

### Life cycle assessment framework

A cradle-to-grave LCA was conducted in line with ISO 14040 and 14044 standards [13] to evaluate the environmental impacts of PD treatments. The study was conducted at four European clinical centres: AOU Policlinico di Modena (Modena), University Medical Centre Utrecht (Utrecht), Medical University of Warsaw (Warsaw), Hospital Universitario Fundación Jiménez Díaz, Madrid (Madrid). Each step of the PD pathway, from patient education to treatment and follow-up, was mapped using flow diagrams and verified with clinical staff.

### Peritoneal dialysis modalities considered

Each centre’s actual patient distribution across PD modalities (CAPD, APD, iPD) was modelled to generate a representative year-long treatment profile. Separate models were also developed for each modality individually to assess comparative impacts. The incremental dialysis in Utrecht and Madrid was only incremental CAPD.

### Life cycle inventory (LCI) data collection

Primary data were collected between April and September 2024, including material weights, clinical schedules, equipment energy use, and travel distances. Secondary data from the EcoInvent v3.10 database [14] were used where primary data were unavailable.

Inventory categories included materials, energy, water, and waste.

### Modelling in openLCA and impact assessment

All collected data were modelled in OpenLCA [15], with separate product systems developed for each clinical centre. Background processes, including material production, electricity generation, transportation, and waste treatment were sourced from the EcoInvent v3.10 database [14]. Environmental impacts were assessed using the Environmental Footprint (EF) 3.1 method, focusing on midpoint indicators. Among the 16 impact categories analysed, particular attention was given to climate change (expressed as kg CO₂-equivalents), nonrenewable energy resource use (MJ), and water use (m³ world equivalents). These categories represent life-cycle-based environmental burdens and are distinct from the operational energy and water consumption values collected during the life cycle inventory phase. To facilitate interpretation and highlight the relative significance of each category, normalization was applied using EF 3.1 normalization factors. This process expresses impacts relative to an average European citizen’s annual environmental footprint, thereby enabling a comparative evaluation across impact categories and enhancing the clarity of result prioritization. The modelling method of transport, energy, and water were in supplementary material (SM1).

### Dismantling and weighing components

PD products, including dialysis bags, tubing, and connectors, were dismantled into their individual components at each clinical centre. Each component was weighed using a precision scale and categorized based on material type [e.g. polypropylene(PP), polyethylene (PE), polyvinyl chloride (PVC)] using information mainly from the manufacturer’s technical data sheet and sometimes from different literatures. All the product images and product lists are uploaded on zenodo.org and the link is in the data availability statement (1,2,3,4,5,6,7,8).

### Identification of procedures and pathways

The LCA of the PD pathways was conducted to map the environmental impacts across each step of the patient’s treatment journey at all four clinical centres. This assessment includes patient education, medical evaluations, treatments, and the disposal of consumables. Key activity data categories considered include energy use, water use, patient and staff travel, waste disposal, and procurement of necessary materials. Up to 17 PD clinical pathways were evaluated: education for RRT, surgical evaluation for PD suitability/PD surgical evaluation, patient’s home suitability assessment(only in Modena), presurgery evaluation/preoperative screening, peritoneal catheter placement under local anaesthesia/radiological placement, surgical placement of PD catheter via laparoscopy (not in Warsaw), postcatheter surgical evaluation/mini site change and postsurgical evaluation, training for CAPD, training for APD. PD dialysis treatment, monthly examination/outpatient examination every 6 weeks, peritoneal equilibration test (PET), change of terminal catheter set (not in Utrecht), management of malfunctioning catheter, peritonitis procedure, and catheter removal. Additionally, patient’s home suitability assessment and pretraining for PD were available for Modena, Overall, 17 pathways were evaluated in Modena, 14 in Warsaw and Utrecht and 15 in Madrid.

### Flow diagrams

Flow diagrams were developed to map each stage of the PD treatment process using Microsoft Visio [16] for all four clinical centres. These diagrams captured activities, materials, energy use, and waste for each stage and were verified with clinical staff and dialysis experts to ensure accuracy.

A sample of the first stage of the pathways which is available in all the four clinical centres is the education on RRT. Each clinical centre has its own protocol even for similar pathways as showcased for Utrecht (Fig. S1). Flow diagrams can be found in the link on materials declaration uploaded to Zenodo.org (1,2,3,4,5,6,7,8).

### System boundaries

**Figure 1:**
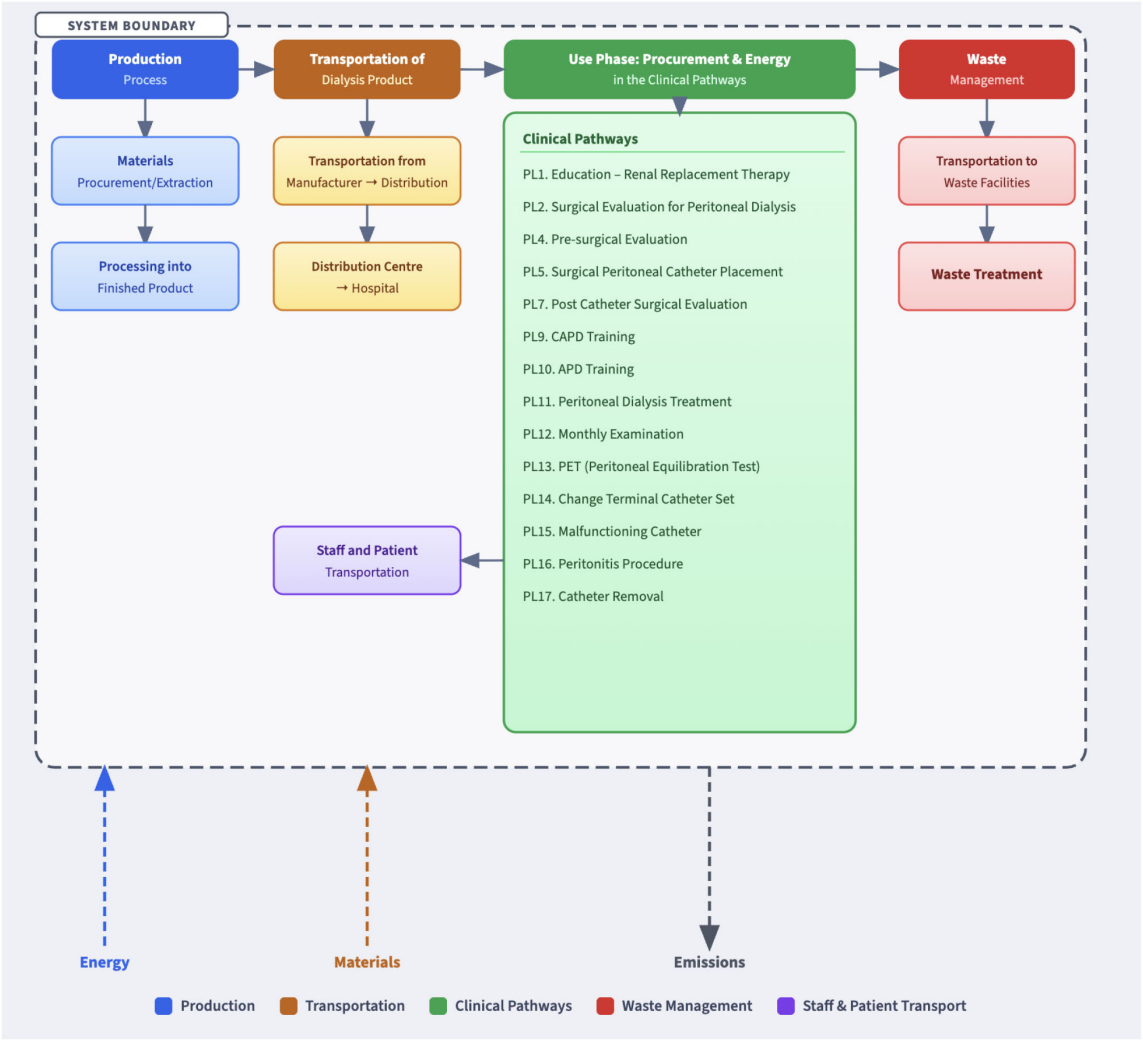
System boundary sample.

The system boundaries (Fig. 1) include material extraction, manufacturing, transportation, usage, and disposal of PD components. Transportation data for patient and staff travel were collected through surveys and interviews across all four clinical centres. Procurement encompasses emissions associated with resources required for material production and processing. Upstream manufacturing processes—including water use associated with plastics production—are captured within the procurement category via EcoInvent v3.10 background datasets.

### Centre characteristics and patient demographics

Table 1 summarizes centre-level characteristics and dialysate prescription data for all four participating centres. The percentage of patients undergoing APD, CAPD, and iPD reflects the actual distribution of these modalities at the time of data collection across the participating centres.

**Table 1:** Centre characteristics and patient demographics.

Parameter	Modena	Utrecht	Warsaw	Madrid
No. patients	65	25	20	40
% on CAPD	54%	12%	60%	60%
% on APD	35%	80%	40%	35%
% on incremental CAPD (iCAPD)	6%	8%	-	5%
% on incremental APD(iAPD)	5%	-	-	-
Mean time on PD (months)	60	60	60	60
Median APD vol/day (l)	10	9.5	10	10
Median CAPD vol/day (l)	6	6	8	4
Median iCAPD vol/day (l)	4	4	-	2
Median iAPD vol/day (l)	5	-	-	-

### Functional unit

The functional unit is defined as 1 year of PD treatment per patient, covering both CAPD and APD modalities. In most clinical centres, iPD was covered.

## RESULTS

### Overall environmental impacts

**Figure 2:**
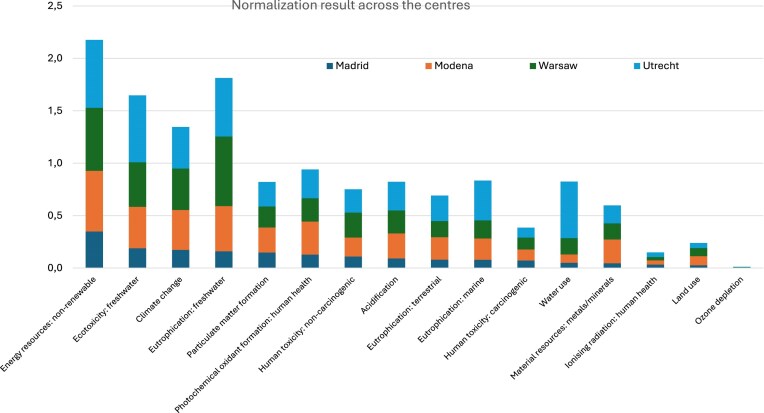
Normalization graph across the four centres.

Environmental impact categories are critical components of environmental assessments. We focused only on midpoint categories, which allow researchers and practitioners to systematically analyse the potential environmental consequences of products and processes across their entire life cycle, from raw material extraction to production, use, and disposal. The comparative LCA revealed notable differences in environmental impacts across the clinical centres, reflecting differences in energy infrastructure, waste management practices, and clinical protocols (Table 2,1). Normalization was performed, and based on the normalized results, the most prominent impact categories across centres were nonrenewable energy use, freshwater ecotoxicity, climate change, and freshwater eutrophication. For detailed analysis, we selected climate change (kg CO₂-eq), nonrenewable energy use (MJ), and water use (m³ world equivalents). The first two were among the top contributors in both absolute and normalized terms, while water use although less dominant overall, was included due to its growing relevance in sustainable healthcare practice and policy, especially considering water scarcity concerns. Table 2 presents the absolute midpoint results for all 16 impact categories across centres, allowing a quantitative comparison of environmental burdens per year of treatment. In contrast, Fig. 2 shows the normalized impacts, providing insight into the relative importance of each category. For readers unfamiliar with LCA terminology, a brief glossary of key impact categories is provided in Supplementary Box S4.

**Table 2:** Life cycle assessment impact categories.

Impact categories	Utrecht	Modena	Poland	Madrid	Unit
Acidification	16.71	13.57	12.82	5.11	mol H+-Eq
Climate change	3319.97	3101.23	3381.14	1736.32	kg CO_2_-Eq
Ecotoxicity: freshwater	30 616.81	25 042.72	25 538.31	11 087.16	CTUe
Energy resources: nonrenewable	54 709.63	40 020.32	47 032.81	28 893.33	MJ, net calorific value
Eutrophication: freshwater	1.20	0.74	1.35	0.26	kg P-Eq
Eutrophication: marine	8.55	4.05	4.29	1.53	kg N-Eq
Eutrophication: terrestrial	47.80	38.62	35.73	14.13	mol N-Eq
Human toxicity: carcinogenic	0.00	0.00	0.00	0.00	CTUh
Human toxicity: noncarcinogenic	0.00	0.00	0.00	0.00	CTUh
Ionising radiation: human health	211.70	172.48	159.21	134.72	kBq U235-Eq
Land use	60 427.14	71 481.42	74 053.66	21 049.31	Dimensionless
Material resources: metals/minerals	0.01	0.01	0.01	0.00	kg Sb-Eq
Ozone depletion	0.00	0.00	0.00	0.00	kg CFC-11-Eq
Particulate matter formation	0.00	0.00	0.00	0.00	disease incidence
Photochemical oxidant formation: human health	12.64	13.13	12.46	5.28	kg NMVOC-Eq
Water use	6631.21	939.81	1348.25	854.22	m3 world Eq deprived

### Global warming potential (GWP)

Global warming potential (GWP) represents the cumulative climate change impact of material procurement, electricity consumption, staff and patient travel, waste treatment, and transport, quantified in CO₂ equivalents (CO₂-eq). To contextualize these figures for a clinical readership: 1200 kg CO2-eq is approximately equivalent to one return long-haul flight from London to New York [17].

There was substantial variability among the four clinical centres. The highest GWP per patient-year was observed in Warsaw (3381.14 kg CO₂-eq), followed closely by Utrecht (3319.97 kg CO₂-eq), Modena (3101.23 kg CO₂-eq), and then Madrid (1736.32 kg CO₂-eq) (Fig. 3A).

**Figure 3:**
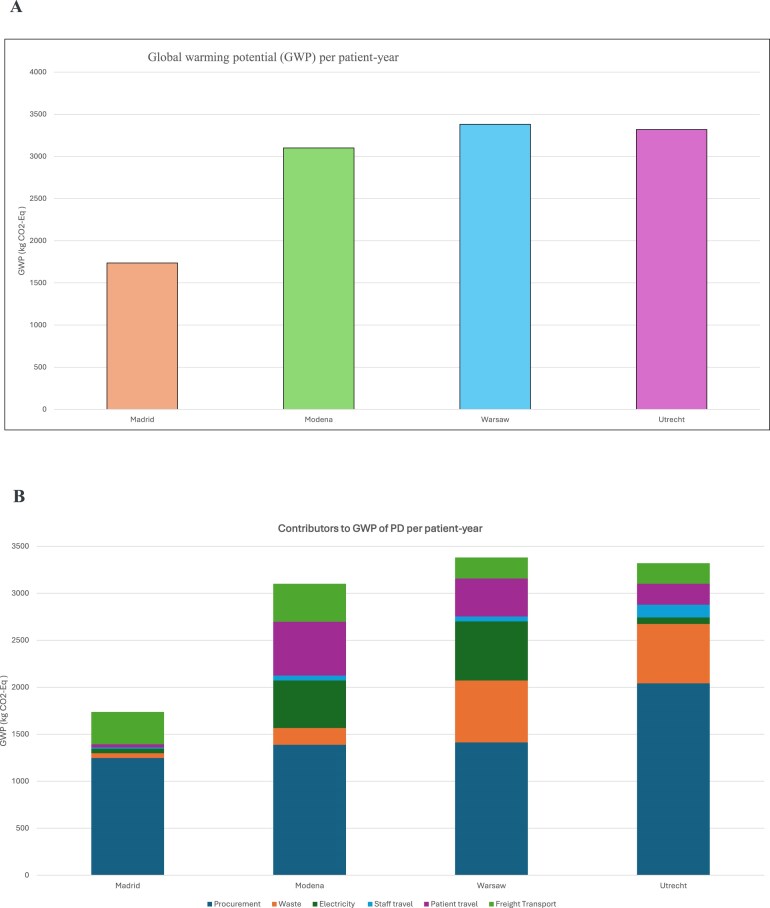
A. Global warming potential (GWP) per patient-year. B. Contributors to GWP of PD per patient-year.C. Nonrenewable energy resources per patient-year. D. Water use per patient-year.

**Figure 3:**
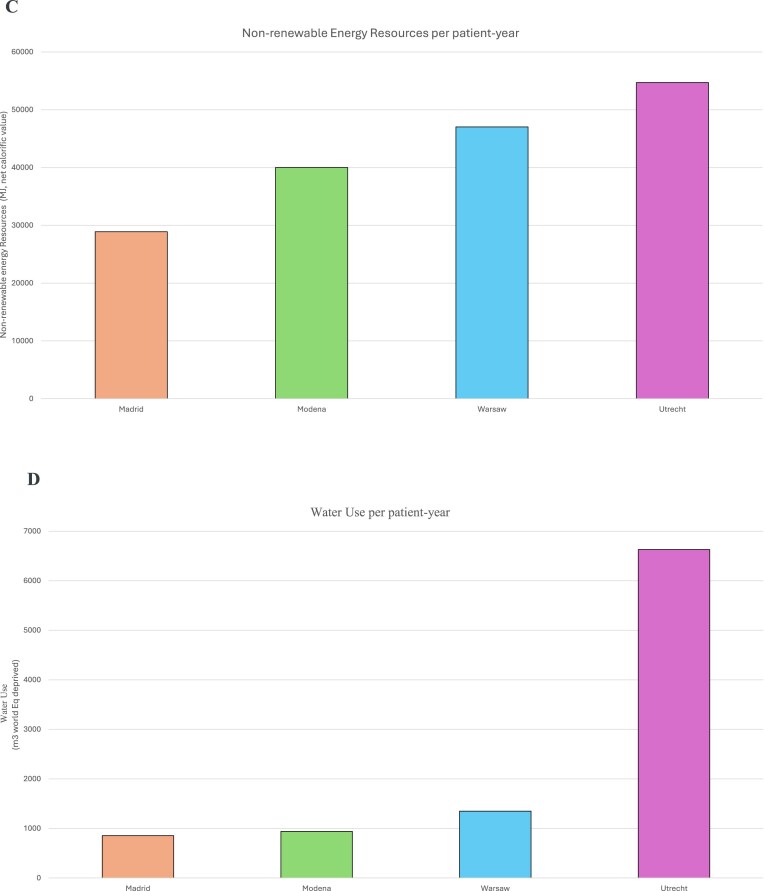
continue

**Figure 4:**
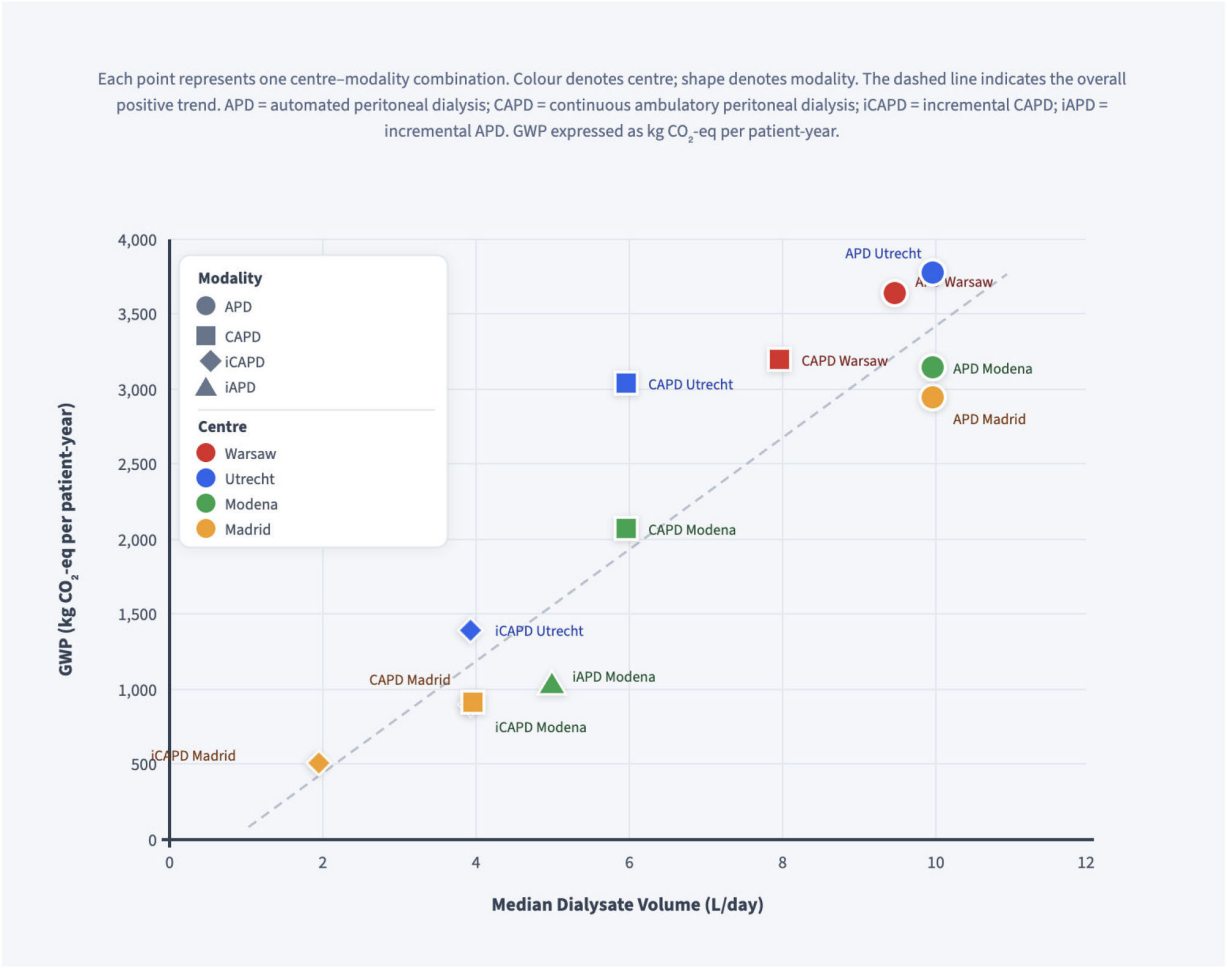
Relationship between median daily dialysate volume and global warming potential (GWP) per patient-year by modality and centre.

A detailed breakdown categorized GWP into six primary contributors: procurement, waste, electricity, staff travel, patient travel, and freight transport (Fig. 3B). There were marked differences in emission profiles between centres. Across all sites, procurement emerged as the principal contributor to total GWP, with Utrecht showing the highest procurement-related emissions (2041 kg CO₂-eq) and Madrid the lowest (1247 kg CO₂-eq). Electricity consumption significantly contributed to emissions in Warsaw and Modena, accounting for 628.46 kg CO₂-eq and 505.52 kg CO₂-eq, respectively. Additionally, staff and patient travel emissions were particularly high in Modena (624.74 kg CO₂-eq) combined. Fig. 4 illustrates the association between prescribed dialysate volume and GWP per patient-year, though considerable variation around this trend reflects the independent contributions of energy sourcing, procurement, and waste management.

### Nonrenewable energy resources

Total cumulative nonrenewable energy resources per patient per year also varied significantly across the centres. Utrecht recorded the highest energy resources at 54 709 MJ, followed by Warsaw (47 032 MJ), Modena (40 020 MJ), and Madrid (28 893 MJ) (Fig. 3C).

### Water use

A critical environmental measure is the water footprint, which quantifies the direct and indirect water use associated with medical procedures. There were substantial differences in water use across the four centres, Utrecht exhibited the highest water use at 6631 m³ per patient-year, followed by Warsaw (1348 m³), Modena (939.8 m³), and Madrid (854 m³) (Fig. 3D).

### APD vs CAPD vs iPD

**Figure 5:**
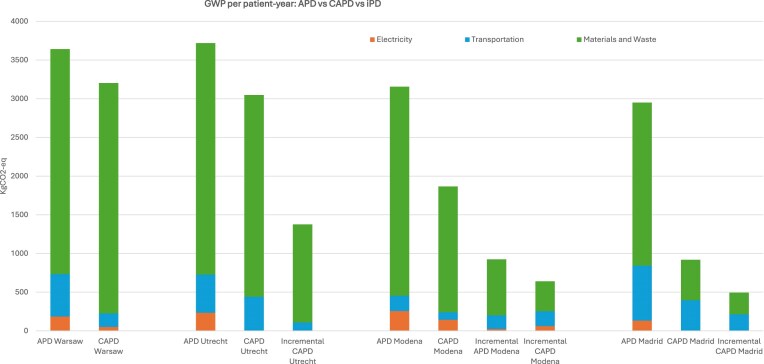
GWP per patient-year; APD vs CAPD vs iPD.

In general, APD demonstrated highest overall environmental impact per patient-year and iPD the lowest, particularly due to reduced dialysate consumption and fewer disposables (Fig. 5). Material use and waste were the primary drivers of emissions in APD and CAPD, followed by electricity (in APD) and transportation (in CAPD).

For APD, the highest GWP was found in Utrecht (3719 kg CO₂-eq), followed by Warsaw and Modena, while Madrid had the lowest. By contrast, the impact of CAPD was very similar in Warsaw, Utrecht and Modena, Warsaw being the highest contributor. Notably, electricity use in APD was substantial in Utrecht and Modena, reflecting high dependence on automated equipment. In contrast, CAPD required less energy but produced more waste from frequent manual exchanges.

There were also differences between the three centres that offered iPD, the environmental impact being highest for Utrecht and lowest for Madrid (494 kg CO₂-eq).

Madrid achieved the lowest GWP for all modalities, benefitting from clean energy and minimal material use.

## DISCUSSION

This study revealed significant variation in the environmental impacts associated with PD across the four clinical centres, allowing the benchmarking of best practice to improve the sustainability of PD. Warsaw and Utrecht exhibited the highest carbon footprints, driven largely by regional factors such as energy sources and extra material use. Conversely, Madrid demonstrated substantially lower environmental burdens across all impact categories. These differences reflect a combination of regional energy infrastructure, procurement practices, waste management policies, and local clinical protocols, emphasizing that the sustainability of PD is strongly context dependent [3, 8]. These findings align with the ERA’s call for centre-specific, evidence-based sustainability assessments that translate into actionable recommendations for nephrologists [2, 3]. All centres' PD carbon footprints in this study align broadly with, or slightly exceed, previously reported ranges (1.4–2.5 t CO₂-eq per patient-year) [3, 8], yet they remain substantially lower than typical emissions associated with HD, which range between 4 and 10 t CO₂-eq per patient-year [3, 17]. A PD-first policy, or more broadly the prioritization of home-based dialysis modalities over in-centre HD where clinically appropriate, likely represents a highly impactful ecological decision, given the substantially lower carbon footprint of PD compared with in-centre HD [3, 8, 18], before considering optimization within PD modalities.

Across all centres, the dominant contributor to environmental burden was the extensive use of single-use plastic consumables, particularly dialysate bags, tubing, and associated packaging. These items, largely manufactured from fossil-based polymers such as PE, PP, and PVC, accounted for the majority of greenhouse gas emissions and cumulative nonrenewable energy demand. Dialysate volume emerged as a key determinant of impact, as CAPD and APD require frequent fluid exchanges throughout the year [18]. Wide international variation in prescribed dialysate volumes—as documented in the Peritoneal Dialysis Outcomes and Practice Patterns Study [19], suggests that prescribing practices represent a significant, modifiable driver of PD’s environmental footprint. Although dialysate volume was a key driver of GWP (Fig. 4), centres with identical prescribed volumes differed by up to 1000 kg CO₂-eq per patient-year, underscoring that volume reduction alone is insufficient and that energy, procurement, and waste management must be addressed in parallel. Consistent with previous PD waste audits, these single-use consumables outweighed the contribution of durable equipment such as cyclers, whose impacts are distributed over many years of use [8, 18, 20]. PD waste audits have shown that a full year of therapy can result in ∼21–27 kg of PP and 81–118 kg of PVC plastic waste per patient, largely due to dialysate packaging and tubing [18]. In CAPD, the high frequency of 2–2.5 l exchanges contributes to a substantial cumulative material weight [21]. Utrecht’s disproportionately high procurement footprint reflects its predominant use of APD (80% of patients vs 35%–40% at other centres), which generates greater total consumable volumes through higher prescribed dialysate loads and APD-specific cassette tubing sets. This finding reinforces that material intensity, rather than device ownership, represents the principal environmental hotspot in PD delivery. With respect to water use, Utrecht’s disproportionately high footprint was primarily driven by cotton gauze consumption, averaging ∼5700 units per patient annually compared with ∼1000 units at other centres. This difference reflects a centre-specific practice of using single-use cotton gauze for wiping surfaces around the patient, whereas other centres employed reusable towels and liquid disinfectant for equivalent environmental control procedures. The agricultural production of cotton is both water- and land-intensive [22], making even lightweight items environmentally significant when used at scale. Other ancillary consumables, including masks, were captured in the inventory across all centres; variation between centres was comparatively modest and did not materially alter the overall results. Similarly, complication-related pathways, including peritonitis management and catheter malfunction, were included at centre-specific frequencies; differences in complication rates between centres were small and contributed minimally to the overall variation in environmental impact observed.

Energy sourcing further shaped centre-specific outcomes. In Warsaw, the carbon intensity of PD was amplified by the fossil-dominated electricity mix, increasing emissions associated with electricity use across PD pathways, particularly for APD where automated equipment is used routinely [23]. In contrast, Madrid’s substantially lower electricity-related emissions reflect the high penetration of renewable energy in Spain’s power grid [23]. These results highlight the sensitivity of PD environmental performance to regional electricity infrastructure.

Waste management practices also played a critical role in differentiating centres. In Warsaw, the routine classification of PD disposables as hazardous waste led to widespread incineration, contributing disproportionately to greenhouse gas emissions, energy use, and water consumption [23]. Long-distance transportation of PD fluids and packaging from the manufacturer further increased the overall footprint. Modena represented an intermediate case, where environmental impacts were driven primarily by logistics and upstream material sourcing rather than electricity use. In contrast, Madrid demonstrated that optimized waste segregation, limited incineration, and partial recycling of noncontaminated materials substantially reduce waste-related impacts [24].

Emissions from patient and staff travel were notably lower in Madrid, primarily due to shorter average patient travel distances to the clinic compared with the other centres, as well as less frequent outpatient visits. In Madrid, routine outpatient follow-up typically occurred once every two months, compared with every 6 weeks in Utrecht and monthly visits in Warsaw and Modena.

Comparison across modalities showed that APD generally exhibited higher environmental impacts than CAPD, driven by greater material use and additional electricity demand. iPD, when clinically appropriate, consistently resulted in the lowest environmental impacts due to reduced dialysate volumes and fewer consumables. This aligns with existing clinical and environmental evidence indicating that iPD can lower resource use while maintaining treatment adequacy, supporting its consideration as a sustainability-oriented PD strategy [25, 26]. A recent cohort study further corroborated these findings, demonstrating that iPD is associated with significantly lower carbon emissions, water use, and dialysis waste compared with full-dose PD [27].

**Figure 6:**
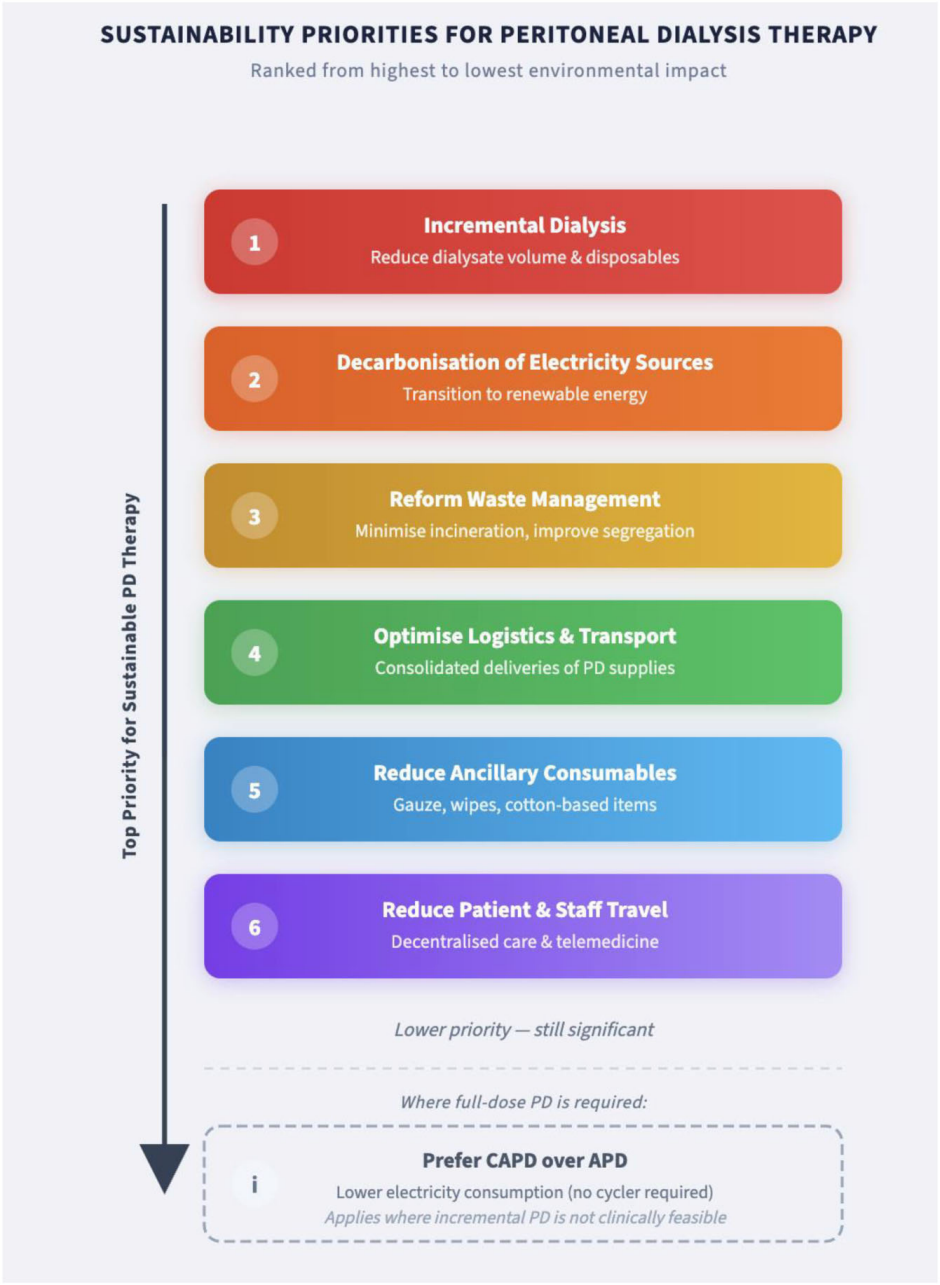
Hierarchy of priority sustainability interventions for PD pathways.

Several opportunities for improving the environmental sustainability of PD pathways emerge from this analysis. Reducing dialysate volume and associated single-use consumables, particularly through the use of incremental peritoneal dialysis (iPD) where clinically appropriate, represents the highest-impact intervention. More broadly, reducing reliance on virgin single-use plastics through material substitution, product redesign, or circular procurement strategies constitutes a key leverage point for impact reduction [20]. Centres operating in regions where the primary source of energy is fossil related would further benefit from transitioning to renewable electricity supplies or improving the energy efficiency of PD equipment, particularly for automated modalities [22, 28]. Waste management reforms, including improved waste segregation and reduced incineration of nonhazardous PD waste streams, offer additional potential for environmental benefit [23]. By segregating waste, we can save around 550 kg of carbon emission equivalent to one-way flight from London to New York. Transport-related emissions may be mitigated through localized procurement, consolidated deliveries, reduced patient and staff travel, and the use of telemedicine where clinically feasible [29, 30]. The relative hierarchy of these improvement opportunities is illustrated in Fig. 6. Importantly, many of these interventions align with improved patient convenience and system efficiency, indicating that environmental sustainability and quality of care are not competing objectives [3].

Some limitations should be acknowledged when interpreting these findings. Certain inventory data, such as water use for hand hygiene, sanitizer consumption, and time allocation for specific procedures, were estimated due to constraints in primary data collection. Background datasets may not fully capture local technologies, and allocation of shared resources required assumptions. Additionally, the analysis reflects a single year of treatment and may not represent longer-term variability in practice. Remote APD monitoring was used at all four centres; however, staff time and energy use associated with data infrastructure were not included within the system boundary, representing a gap that future studies should address as digital health infrastructure becomes more widely adopted. It is important to note that the sustainability interventions proposed here, including reduced dialysate volumes, material substitution, and modality choice must be considered alongside clinical effectiveness. Formal comparison of peritonitis rates, exit site infection rates, and technique survival across centres was outside the scope of this environmental LCA. Future work integrating clinical outcome data with environmental impact assessments would allow a more complete evaluation of value-based sustainability in PD care. Nonetheless, with respect to the environmental analysis itself, the use of standardized LCA methods, transparent assumptions, and consistent system boundaries across centres strengthens the robustness of the comparative conclusions [5, 13].

Overall, this study demonstrates that the environmental footprint of PD varies across European clinical centres, driven primarily by material consumption, energy sourcing, waste management, and care delivery models. Procurement of dialysate fluids and single-use consumables emerged as the dominant environmental hotspot, while renewable energy use, optimized waste practices, and reduced material intensity enabled substantially lower impacts. These findings indicate that environmentally sustainable PD is achievable through targeted, context-specific interventions that align environmental stewardship with high-quality, patient-centred care [3, 8].

## Supplementary Material

sfag156_Supplemental_File

## Data Availability

All environmental inventory and modelling data generated or analysed during this study are publicly available in the following Zenodo repository: 1. Fehintola, A., Larkin, J., Martinez, R., & Duane, B. (2025). Product Images of Life Cycle Assessment Dataset For Peritoneal Dialysis in Utrecht, Netherlands [Data set]. Zenodo. https://doi.org/10.5281/zenodo.14601573 2. Fehintola, A., Larkin, J., Martinez, R., & Duane, B. (2024). Life Cycle Assessment Dataset For Peritoneal Dialysis in Utrecht, Netherlands [Data set]. Zenodo. https://doi.org/10.5281/zenodo.14601606 3. Fehintola, A., Larkin, J., Martinez, R., & Duane, B. (2024). Product Images of Life Cycle Assessment Dataset For Peritoneal Dialysis in Warsaw, Poland [Data set]. Brett Duane. https://doi.org/10.5281/zenodo.14273031 4. Fehintola, A., Larkin, J., Martinez, R., & Duane, B. (2024). Life Cycle Assessment Dataset For Peritoneal Dialysis in Warsaw, Poland [Data set]. Brett Duane. https://doi.org/10.5281/zenodo.14535337 5. Larkin, J. (2024) ‘Life Cycle Assessment Dataset For Peritoneal Dialysis in Modena’. Brett Duane. doi: 10.5281/zenodo.14258866 6. Larkin, J. (2024) ‘Product Images for Life Cycle Assessment Dataset For Peritoneal Dialysis in Modena’. Brett Duane. doi: 10.5281/zenodo.14258920 7. Martínez, R. (2024) ‘Life Cycle Assessment Dataset For Peritoneal Dialysis in Madrid’. Brett Duane. https://zenodo.org/records/14274867 8. Martínez Cadenas, R., Larkin, J., Fehintola, A., & Duane, B. (2024). Product Images of Life Cycle Assessment Dataset For Peritoneal Dialysis in Madrid, Spain (Version v1) [Data set]. Zenodo. https://doi.org/10.5281/zenodo.14274312
