# Supplementary material for: Environmental footprint of peritoneal dialysis in Europe: a comparative life cycle assessment across four European centres
Source: Clin Kidney J. 2026 May 13;19(6):sfag156. doi: 10.1093/ckj/sfag156 (PMC13223597; doi:10.1093/ckj/sfag156)
Supplement: sfag156_Supplemental_File [file sfag156_supplemental_file.docx]

**Supplementary Figures**

**Figure S1: Sample of education on renal replacement therapy pathway in Utrecht**


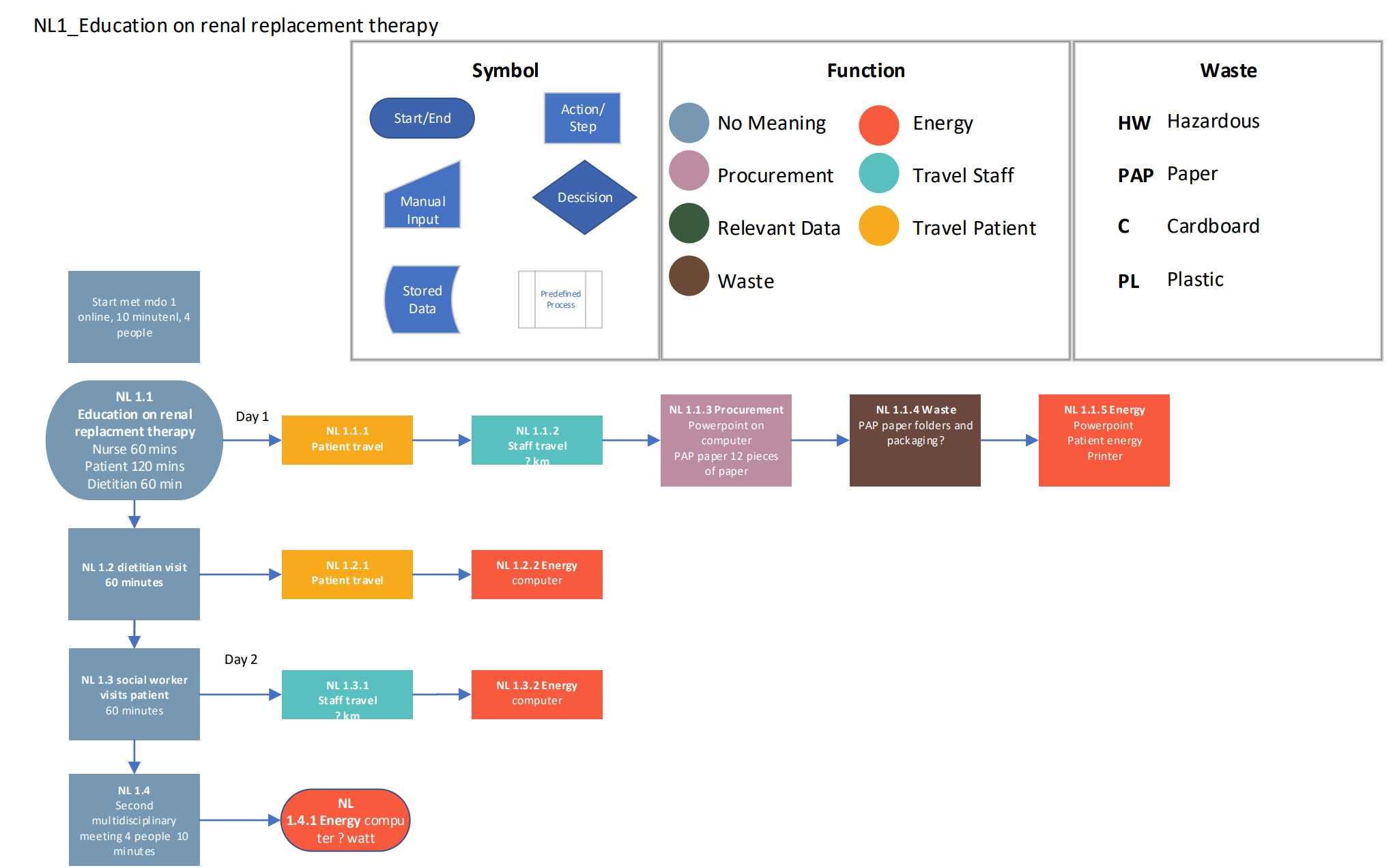


**Figure S2: Water contribution graph at Madrid and Utrecht**

**
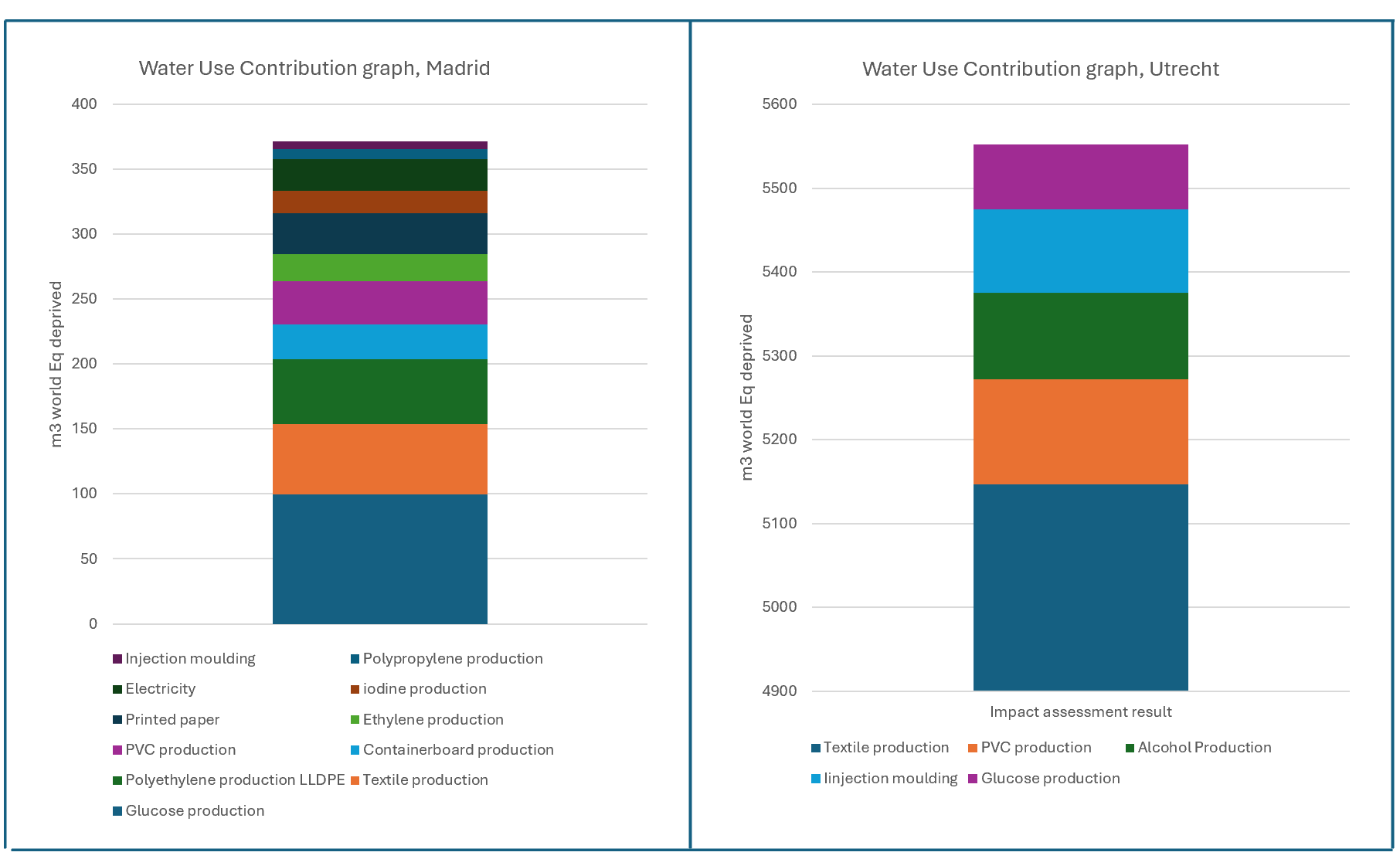
**

**Figure S3:** **Water contribution graph at Modena and Warsaw**

**
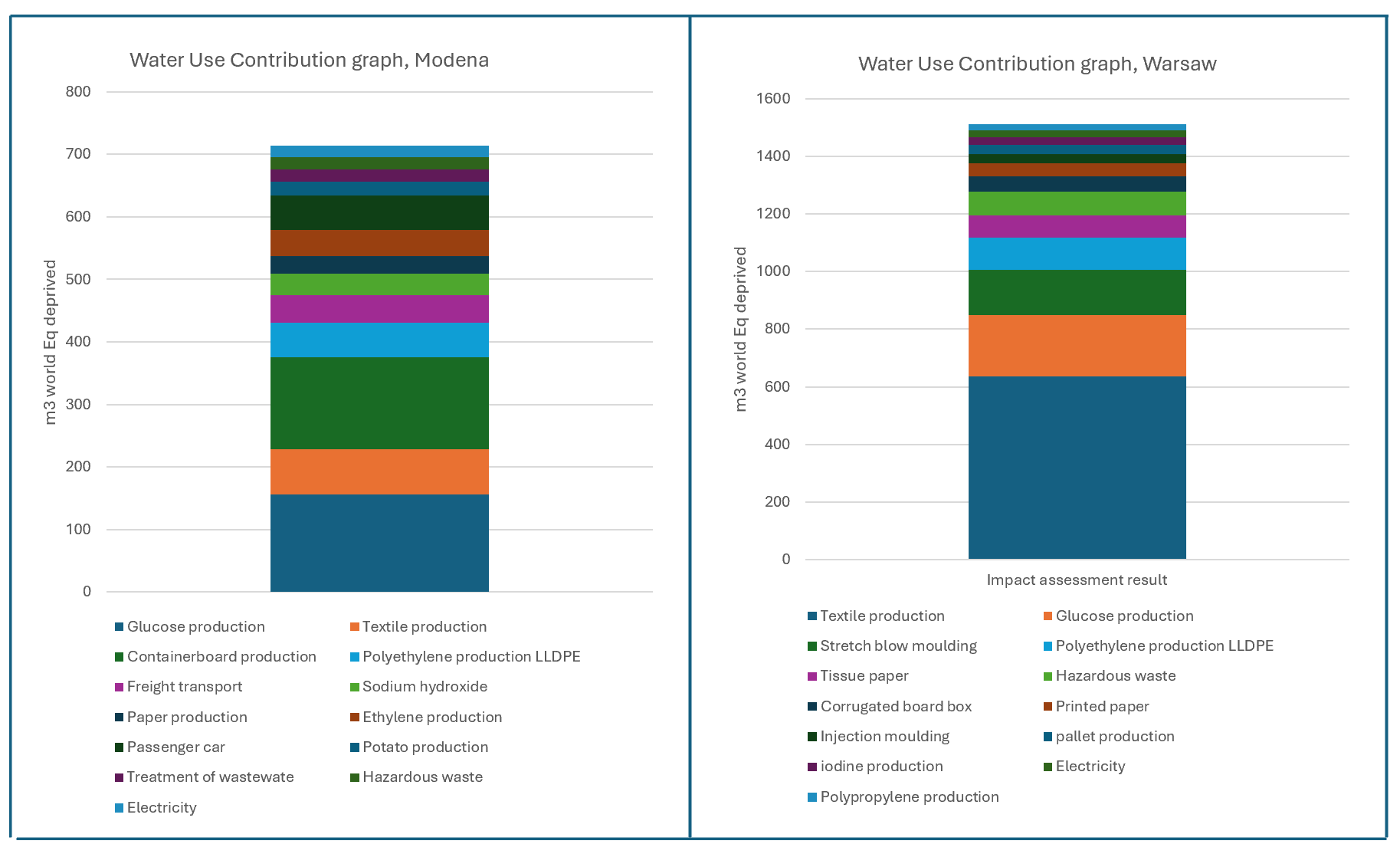
**

**Supplementary Tables**

**Table S1. GWP of different PD modalities. Same data are show in graphic form in figure 5**

| Clinical Centres | Electricity | Transportation | Materials and Waste | Total (Kg CO_2_ eq) |
| --- | --- | --- | --- | --- |
| Warsaw |  |  |  |  |
| APD | 185.6 | 549.4 | 2906 | 3641 |
| CAPD | 50 | 175 | 2977 | 3202 |
|  |  |  |  |  |
| Utrecht |  |  |  |  |
| APD | 233 | 495 | 2991 | 3719 |
| CAPD | 0 | 441 | 2607 | 3048 |
| iPD | 0 | 109 | 1267 | 1376 |
| Modena |  |  |  |  |
| APD | 255.8 | 197.6 | 2701.8 | 3155.2 |
| CAPD | 142.8 | 97.4 | 1626.2 | 2069.6 |
| iPD APD | 24 | 176.6 | 723 | 1024.2 |
| iPD CAPD | 62.2 | 190.8 | 386.6 | 886.8 |
| Madrid |  |  |  |  |
| APD | 132 | 711 | 2107 | 2950 |
| CAPD | 0 | 396 | 523 | 919 |
| iPD | 0 | 213 | 281 | 494 |

**SM1: Supplementary Materials**

## Transportation Modelling

Patient and staff travel emissions were modelled using clinical schedules, travel logs, and interviews. Mode of transport, distance, and visit frequency were collected and converted to emissions using regional factors. Staff emissions were allocated based on time spent per procedure rather than evenly across patients.

## Operational Energy Consumption

Energy use was categorized into facility-level consumption and equipment-specific usage as facility and device energy. Facility energy refers to electricity and heating used in dialysis wards were derived from hospital bills and adjusted based on floor area and patient throughput. Device energy refers to energy consumption of medical equipment was calculated from technical specifications, usage frequency, and duration of use during PD treatments.

To reflect regional differences in power generation, energy consumption data were linked to country-specific electricity mixes in the modelling stage.

## Water Consumption Values

Water consumption was assessed by combining direct consumption (e.g., handwashing, cleaning) with indirect water use embedded in PD fluid and consumable production. Facility-level data were allocated based on patient activity or treatment time. Water used in PD fluid production was included via upstream modelling, ensuring that the total water footprint captured both direct and indirect contributions.

## Inputting Data into OpenLCA

Data collected on material weights, flow diagrams, and processes were input into OpenLCA software. Processes were modelled for each clinical centre, and environmental impacts were assessed using the EcoInvent database.

## Life Cycle Inventory (LCI) Data Collection

To assess materials, all disposables (e.g., bags, gloves, tubing) were dismantled and weighed by material type. For energy, facility-level energy data were allocated based on patient volume and space. Device-level usage was calculated using power ratings and usage durations. For water, direct use (e.g., handwashing) was estimated by clinical staff, while embedded water in product manufacturing was included via upstream modelling. Waste streams were categorised (plastic, paper, general, healthcare) and linked to appropriate disposal processes per country.

All inputs and outputs were normalised to the functional unit.

## Modelling in OpenLCA and Impact Assessment

For material procurement, production processes were geographically customized based on supplier origin. For example, Baxter fluid bags, which were primarily manufactured in Ireland, were modelled using Irish electricity and material inputs. The transportation of these products from the production site to each clinical centre was also accounted for, using appropriate freight transport modes and distances.

**Supplementary Box S4**

**Glossary of Life Cycle Assessment (LCA) Impact Categories**

*A reference guide for readers unfamiliar with environmental science terminology*

Life cycle assessment (LCA) evaluates the environmental impact of a product or process across its entire lifespan from raw material extraction through manufacturing, use, and disposal. Results are expressed across multiple "impact categories", each measuring a different type of environmental harm. The table below defines each category used in this study, explains what it measures in plain language, and provides a clinically relevant example to aid interpretation.

| **Impact Category** | **What it measures (plain language)** | **Clinical/dialysis example** |
| --- | --- | --- |
| **Climate Change / Global Warming Potential (GWP) Unit: kg CO₂-eq** | The total greenhouse gas emissions associated with a product or process, expressed as CO₂ equivalents. This is the most widely recognised environmental metric. A useful benchmark: 1,000 kg CO₂-eq ≈ one return long-haul flight (e.g., Dublin to New York). | *Burning fossil fuels to power APD cyclers, manufacturing and transporting plastic dialysate bags, freight transport of PD supplies from manufacturer to hospital, patient and staff travel to dialysis centres, and incinerating PD waste all contribute to GWP.* |
| **Non-renewable Energy Use Unit: MJ (megajoules)** | The total amount of fossil fuel energy consumed across the life cycle, coal, oil, and natural gas. This is distinct from electricity use alone; it captures the energy embedded in manufacturing, transport, and waste treatment as well. | *Producing polypropylene and PVC for dialysate bags and tubing requires large amounts of fossil fuel energy, even before the product reaches the patient.* |
| **Water Use Unit: m³ world equivalents (deprived)** | A scarcity-weighted measure of water consumption. it accounts not just for how much water is used, but for how scarce that water is in the region where it is consumed. Water use in arid regions counts more than in water-rich regions. | *Upstream plastics manufacturing (for dialysate bags and tubing) and electricity generation contribute a baseline water footprint common to all centres. Cotton gauze production is additionally highly water-intensive). Utrecht's disproportionately high use of cotton gauze (~5,700 units/patient/year vs ~1,000 at other centres) was the primary driver of its exceptionally elevated water footprint (6,631 m³ vs 570–1,348 m³ at other centres).* |
| **Eutrophication Freshwater Unit: kg P-eq (phosphorus equivalents)** | Excess nutrients (especially phosphorus) entering freshwater bodies such as rivers and lakes, causing algal blooms and oxygen depletion that kill aquatic life. Often caused by agricultural runoff or industrial wastewater. | *Production of plastics and packaging for dialysis consumables releases phosphorus-containing compounds into waterways during manufacturing.* |
| **Eutrophication Marine Unit: kg N-eq (nitrogen equivalents)** | Similar to freshwater eutrophication but occurring in marine (coastal and ocean) environments. Excess nitrogen is the primary driver. Can create "dead zones" where oxygen levels are too low to support marine life. | *Nitrogen oxide emissions from freight transport of PD fluids and supplies contribute to marine eutrophication.* |
| **Eutrophication Terrestrial Unit: mol N-eq** | The deposition of excess nitrogen onto land ecosystems, altering soil chemistry and reducing biodiversity. Caused primarily by nitrogen oxide (NOx) and ammonia (NH₃) emissions from combustion and agriculture. | *Vehicle emissions from freight transport, also patient and staff travel to dialysis centres contribute to terrestrial eutrophication.* |
| **Acidification Unit: mol H⁺-eq** | The release of acid-forming pollutants — primarily sulphur dioxide (SO₂) and nitrogen oxides (NOx) — into the atmosphere. These return to earth as acid rain, damaging forests, soils, freshwater ecosystems, and buildings. | *Coal-dominated electricity grids (such as Poland's) produce high SO₂ and NOx emissions, making electricity-intensive APD more acidifying in Warsaw than in Madrid.* |
| **Ecotoxicity Freshwater Unit: CTUe (comparative toxic units)** | The potential for chemicals released during production, use, or disposal to harm freshwater organisms (fish, invertebrates, algae). Measured as the fraction of species exposed to harmful concentrations. | *Metal contaminants and plastic additives released during manufacturing of dialysis equipment can accumulate in freshwater ecosystems.* |
| **Human Toxicity Carcinogenic Unit: CTUh** | The potential for chemicals to cause cancer in humans, integrated over the whole population exposed. Includes heavy metals, dioxins, and certain organic compounds released during manufacturing or incineration. | *Incineration of PVC-containing PD waste (as practised in Warsaw) can release dioxins and other carcinogenic compounds.* |
| **Human Toxicity Non-Carcinogenic Unit: CTUh** | The potential for chemicals to cause non-cancer health effects (e.g., neurological, reproductive, or developmental harm) in humans. Includes heavy metals such as lead and mercury. | *Metal processing during manufacture of cycler components and connectors may release non-carcinogenic toxic compounds.* |
| **Particulate Matter Formation Unit: disease incidence** | The generation of fine airborne particles (PM2.5 and PM10) or their gaseous precursors, which when inhaled cause respiratory and cardiovascular disease. Expressed as estimated cases of disease per unit of output. | *Diesel freight vehicles transporting PD fluids from manufacturers to distribution centres are a key source of particulate matter.* |
| **Photochemical Oxidant Formation (human health) Unit: kg NMVOC-eq** | The formation of ground-level ozone and other photochemical smog components from reactions between sunlight, nitrogen oxides, and volatile organic compounds. Causes respiratory irritation and reduced lung function. | *Emissions from vehicle exhausts, patient travel, staff travel, and freight contribute to photochemical smog formation.* |
| **Ionising Radiation Unit: kBq U235-eq** | Exposure of humans to ionising radiation from nuclear power generation in the background energy mix. Countries with higher nuclear energy penetration (e.g., France) show higher scores on this metric even though nuclear power has low GWP. | *The electricity mix in each country determines ionising radiation scores, a trade-off with low-carbon nuclear power.* |
| **Land Use Unit: dimensionless (Pt)** | The occupation and transformation of land for agriculture, industry, or infrastructure, weighted by the quality of the land affected. Intensive land use reduces biodiversity and disrupts ecosystem services. | *Cotton farming for gauze, mask,etc production and agriculture for bio-based packaging materials are land-use intensive. Utrecht's high gauze consumption contributes to its land use score.* |
| **Material Resources Metals/Minerals Unit: kg Sb-eq (antimony equivalents)** | The depletion of scarce metallic and mineral resources used in manufacturing. Expressed relative to the scarcity of antimony (Sb) as a reference material. | *Electronic components in APD cyclers and connectors contain rare metals (e.g., copper, cobalt). Their scarcity is captured in this category.* |
| **Ozone Depletion Unit: kg CFC-11-eq** | The destruction of stratospheric ozone by halogenated compounds (e.g., CFCs, HCFCs), which protects Earth from harmful UV radiation. Largely controlled by the Montreal Protocol but still relevant for industrial processes. | *Certain refrigerants and aerosol propellants used in manufacturing or cold-chain logistics may contribute marginally to ozone depletion.* |

**A note on normalisation**

To allow meaningful comparison across these diverse impact categories, results in this study were normalised using Environmental Footprint (EF) 3.1 factors. Normalisation expresses each impact as a fraction of the average European citizen's annual environmental footprint for that category. For example, a normalised climate change score of 0.5 means the treatment contributes half the annual per-capita European carbon footprint. This allows the reader to see at a glance which impact categories are most environmentally significant relative to everyday human activity.

*Source note: Definitions adapted from the European Commission Environmental Footprint (EF) 3.1 methodology and ISO 14044:2006 Life Cycle Assessment standard. CFC = chlorofluorocarbon; CTUe = comparative toxic unit for ecosystems; CTUh = comparative toxic unit for humans; GWP = global warming potential; kBq = kilobecquerel; LCA = life cycle assessment; MJ = megajoule; NMVOC = non-methane volatile organic compound; NOx = nitrogen oxides; PM = particulate matter; PVC = polyvinyl chloride; SO₂ = sulphur dioxide.*
